# The Comparison of Ultrasound and Tomographic Images of Lung Involvement in Critically Ill Patients With COVID-19 Pneumonia: A Prospective Observational Study

**DOI:** 10.7759/cureus.58201

**Published:** 2024-04-13

**Authors:** Funda GOK, Korhan Kollu, Necdet Poyraz, Hulya Vatansev, Alper Yosunkaya

**Affiliations:** 1 Department of Critical Care Medicine, Necmettin Erbakan University, Meram School of Medicine, Konya, TUR; 2 Department of Critical Care Medicine, Konya City Education and Research Hospital, Konya, TUR; 3 Department of Radiology, Necmettin Erbakan University, Meram School of Medicine, Konya, TUR; 4 Department of Pulmonology, Necmettin Erbakan University, Meram School of Medicine, Konya, TUR

**Keywords:** critically ill patients, lung imaging, computed tomography, lung ultrasonography, covid-19 pneumonia

## Abstract

Introduction

Computed tomography (CT) has a high sensitivity for diagnosing COVID-19 pneumonia in critically ill patients, but it has significant limitations. Lung ultrasonography (LUS) is an imaging method increasingly used in intensive care units. Our primary aim is to evaluate the relationship between LUS and CT images by scoring a critically ill patient who was previously diagnosed with COVID-19 pneumonia and underwent CT, as well as to determine their relationship with the patient's oxygenation.

Methods

This was a single-center, prospective observational study. The study included COVID-19 patients (positive reverse transcription polymerase chain reaction, RT-PCR) who were admitted to the intensive care unit between June 2020 and December 2020, whose oxygen saturation (SpO_2_) was below 92%, and who underwent a chest tomography scan within the last 12 hours. CT findings were scored by the radiologist using the COVID-19 Reporting and Data System (CO-RADS). The intensivist evaluated 12 regions to determine the LUS score. The ratio of the partial pressure of oxygen in the arterial blood to the inspiratory oxygen concentration (PaO_2_/FiO_2_) was used to assess the patient's oxygenation.

Results

The study included 30 patients and found a weak correlation (ICC = 0.45, 95% CI = 0.25-0.65, p < 0.05) between total scores obtained from LUS and CT scans. The correlation between the total LUS score and oxygenation (r = -0.514, p = 0.004) was stronger than that between the CT score and oxygenation (r = -0.400, p = 0.028). The most common sonographic findings were abnormalities in the pleural line, white lung, and subpleural consolidation. On the other hand, the CT images revealed dense ground-glass opacities and consolidation patterns classified as CO-RADS 5.

Conclusion

A weak correlation was found between LUS and CT scores in critically ill COVID-19 pneumonia patients. Also, as both scores increased, oxygenation was detected to be impaired, and such a correlation is more evident with the LUS score.

## Introduction

The chest computed tomography (CT) scan is a gold standard imaging method to assess the severity of pneumonia in patients infected with coronavirus disease-19 (COVID-19) [1]. The sensitivity of chest CT is 97% for COVID-19 pneumonia [2]. The most obvious limitation of chest CT as a diagnostic tool is the transfer of the patient with respiratory distress to the radiology unit. This procedure also increases the risk of infection transmission [3]. For this reason, lung ultrasonography (LUS), which can be performed and has the characteristic of repetition at the bedside, comes to the forefront as an alternative method to CT [4].

LUS examination can easily detect common pathologies in COVID-19 pneumonia, including dependent region consolidations, subpleural consolidations, alveolar edema, and interstitial thickening. As a result, LUS and CT images have been compared in numerous studies at different stages of COVID-19 [4-7]. In some studies, the B-line score was used, while in others, only subpleural consolidations were looked at [4,8].

Our primary objective is to establish the relationship between LUS and CT findings in a critically ill patient who had already been diagnosed with COVID-19 pneumonia (positive reverse transcription polymerase chain reaction, RT-PCR) and had undergone a CT scan within the last 12 hours. For this purpose, it is aimed at performing LUS examinations by using both linear and convex probes together. This method was used to classify COVID-19 LUS findings [4]. However, the number of studies using this method to compare LUS and CT findings in COVID-19 pneumonia is rare. The secondary aim was to evaluate the correlation between imaging scores and oxygenation levels.

## Materials and methods

The present study was conducted as a prospective observational single-center study in the intensive care unit (ICU) of a university hospital between June 1, 2020, and December 31, 2020. The Local Ethics Committee approved the study protocol (2020/2535). Written informed consent was obtained from COVID-19 patients or their relatives. This research was carried out in compliance with the most recent Helsinki Declaration standards. The study included individuals aged 18-80 years with proven severe acute respiratory syndrome coronavirus-2 (SARS-CoV-2) infection using polymerase chain reaction (PCR) testing. Patients admitted to the ICU due to shortness of breath or low oxygen saturation (less than 92% SpO_2_) and who had undergone a chest CT scan within the last 12 hours were enrolled in the study. It was intended that the interval between the chest CT and LUS examination should not exceed 12 hours. Upon admission to the ICU, the patient had already undergone a chest. The LUS examination was performed subsequently in the ICU. However, if the period between CT and LUS was more than 12 hours, participants were excluded from the study. In addition, patients with a body mass index (BMI) of more than 40, chronic obstructive pulmonary disease, interstitial lung disease, and class IV heart failure according to the New York Heart Association functional classification were excluded from the study. The patients were evaluated immediately upon admission to the ICU by sonography.

Demographic characteristics, systemic diseases, and onset time of symptoms of the patients admitted to the ICU were noted. The type of oxygen therapy used (high-flow nasal cannula [HFNC], noninvasive or invasive mechanical ventilation) was noted, the levels of FiO_2_ and PaO_2_/FiO_2_ were calculated, and the LUS examination was carried out, as explained below. LUS examination was performed by an intensivist who was highly experienced in LUS and blinded to CT findings (F.G.). Each hemithorax was divided into anterior, lateral, and posterior regions using the anterior and posterior axillary lines. Then, each region was further separated into upper and lower areas at the level of the third intercostal space, resulting in a total of 12 areas. The sonographic examinations were conducted employing both convex and linear probes. Initially, a comprehensive assessment of the lungs was performed, with a focus on artifacts associated with LUS parenchyma, using a convex probe. Subsequently, with a linear probe, pleural images and subpleural consolidations were examined. LUS scoring was carried out according to the following criteria [4,7].

Score 0: The pleural line is regular. The lung is sliding, A-lines are present, and there are two or fewer of these A-lines than B-lines.

Score 1: The pleural line is indented, and there are three or more B-lines.

Score 2: The pleural line is broken at many points, and there are small consolidated areas under the pleura. The B-lines associated with these consolidated areas form a white lung image.

Score 3: There are large, consolidated areas under the pleura. The score and total score obtained from each hemithorax were recorded. Additionally, in the examination conducted with a convex probe, the amount of pleural effusion was subjectively evaluated and classified as minimal, moderate, or severe. A highly experienced radiologist in thoracic radiology who was blinded to clinical information independently reviewed the tomography images (N.P.). The chest CT images were assessed and classified under the COVID-19 Reporting and Data System (CO-RADS) score [9]. Visual CT scores were determined based on the CT images, considering the extent and distribution of ground glass opacities and consolidations.

Statistical analysis

All statistical analyses were performed with the Statistical Package for Social Sciences (SPSS) package for Windows, Version 25.0 (Armonk, NY, USA). The findings were presented as means and standard deviations (SD) for numerical variables, while the frequencies and percentages were given for categorical data. The compatibility of numerical variables with a normal distribution was evaluated using the Shapiro-Wilk test. The degree of concordance between the LUS score and CT score was evaluated using the intraclass correlation coefficients (ICC). An ICC below 0.50 indicated poor agreement, between 0.50 and 0.75 indicated moderate agreement, between 0.75 and 0.90 indicated good agreement, and between 0.90 and 1 indicated excellent agreement. The correlations between oxygenation level and imaging scores were evaluated with Spearman's rank correlation coefficient. A p-value of <0.05 was considered sufficient for statistical significance.

## Results

**Figure 1:**
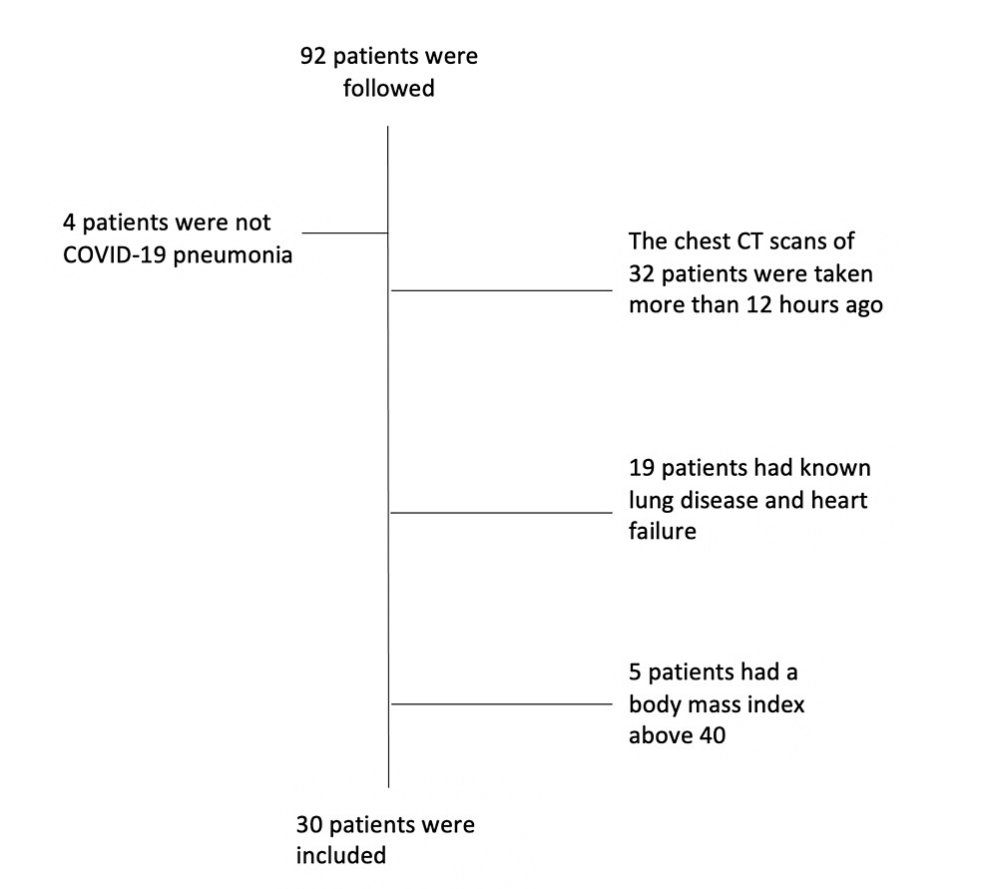
Flowchart of patient selection.

A total of 92 COVID-19 patients were followed up between the specified dates in our ICU. Some patients were excluded from the study, and 30 patients were included in the study (Figure 1).

The mean age of the participants was found to be 65.2 ± 14.4 years. The most common comorbidities were hypertension, 46.7%, and diabetes mellitus, 23.3% (Table 1).

**Table 1:** Participant’s characteristics. SD, standard deviation; BMI, body mass index; SOFA, Sequential Organ Failure Assessment; APACHE, Acute Physiologic Assessment and Chronic Health Evaluation Scoring System; ICU, intensive care unit

	Mean ± SD
Age	65.2 ± 14.4
BMI	26.7 ± 4.1
Male/female (%)	60/40
SOFA score (mean ± SD)	5.8 ± 2.6
APACHE-II (mean ± SD)	15.5 ± 7.9
ICU length of stay (day)	16.1 ± 12.8
Hospital length of stay (day)	21.3 ± 14.2
Mortality (%)	56.7

**Figure 2:**
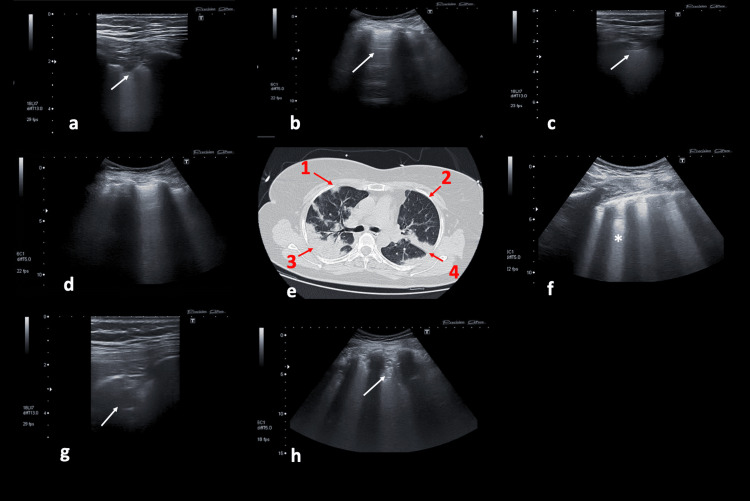
The images of LUS and CT of the patients with COVID-19 pneumonia The a-d, g-h, and c-f are the examinations of the same area via a different probe. (a, b, c, d, f, g, h) LUS. (e) CT. a. Subpleural consolidation (white arrow) was obtained with the linear probe, LUS score 2, corresponding to area 1 (red arrow) on CT. b. In the image, 'A lines' (white arrow) are present. This image was evaluated as LUS score 0. This image corresponds to area 2 on CT. c. The normal appearance of the pleural line has disappeared and there is major consolidation. This image was evaluated as LUS score 3. It corresponds to area number 4 in CT. d. B lines are more than 3. LUS score 1, corresponding to area 1 on CT. e. CT. g. The consolidation image was examined with a linear probe, corresponding to area 3 on CT. h. The consolidation pattern obtained when examined with a convex probe, LUS score 3, corresponding to area 3 on CT. f. White lung (*) image obtained when examined with a convex probe, LUS score 3, corresponding to area 4 on CT. CT, computed tomography; LUS, lung ultrasonography

A total of 11 patients underwent invasive mechanical ventilation, and the other patients received noninvasive mechanical ventilation and high-flow oxygen therapy. The average rate of PaO_2_/FiO_2_ was also found to be 142 ± 69.7. The LUS findings of the patients were examined in 12 areas in each patient, totaling 360 areas. Pathologies were detected in bilateral lung areas in all cases (Video 1, Video 2, Table 2, Figure 2).

**Video 1:** The LUS image of the patient followed up with COVID-19 pneumonia. B lines are more than 3. LUS score was evaluated as 1. This is the video of Figure 2d. LUS, lung ultrasonography

**Video 2:** The images of LUS and CT of the patients with COVID-19 pneumonia A subpleural consolidation and white lung (*) images were obtained when examined with a convex probe. LUS score was 3. This is the video of Figure 2f. LUS, lung ultrasonography

**Table 2:** The findings of lung ultrasonography of the patients with COVID-19 pneumonia.

	n (%)
Bilateral involvement	30 (100)
Confluent B lines (white lung)	29 (96.6)
B-lines (multiple, well defined)	16 (53.3)
Subpleural consolidation	24 (80)
Nontranslobar or translobar consolidation	17 (56.6)
Pleural effusion	1 (3.3)
Pleural line irregularity	30 (100)
Total lung ultrasonography score	20 ± 5.4

The sonographic findings most frequently encountered included irregular or disrupted pleural lines, confluent B-lines, and subpleural consolidation. The total LUS score was determined to be 20 ± 5.4. It was determined to be 20 ± 3.8 and 19 ± 7.1 in those with invasive mechanical ventilation and noninvasive mechanical ventilation, or HFNC, respectively. No difference in LUS scores was detected among the patients who received either invasive mechanical ventilation or noninvasive oxygen therapy.

Upon analyzing the CT results of COVID-19, it was found that all six lung lobes were affected in 93% of cases. The CT score of the lesions was assessed as CO-RADS 5 in 27 patients. The mean of all involvement and the CT visual score averaged 68 ± 22. The lesions were observed as consolidation in 19 individuals and as ground-glass opacities and consolidations in eight patients (Figure 2e). Considering the distribution, the lesions were peripherally located in 22 patients. Additionally, the lesions were mostly circular and showed an irregular pattern. Atypical findings were determined in three patients; one of these patients had diffuse ground-glass opacities, and the CO-RADS score was evaluated as 3. On the other hand, CO-RADS 2 showed unusual findings in the other two patients, such as septal thickening, atelectasis, and pneumonic consolidation. The correlation between the LUS score and CT score was weak (ICC = 0.45, 95% CI = 0.25-0.65, p < 0.05). Additionally, there was a significant correlation between the total LUS scores and PaO_2_/FiO_2_ (r = -0.514, p = 0.004). Given PaO_2_/FiO_2_, a significant correlation was detected between the total CT scores and PaO_2_/FiO_2_ (r = -0.400, p = 0.028).

## Discussion

This study shows that there is a low level of correlation between sonographic and tomographic images in patients with a diagnosis of COVID-19 pneumonia. These patients, who had a positive PCR test and lung tomography before being admitted to the ICU, were examined with LUS upon admission to the ICU. Additionally, as the LUS and CT scores increased, oxygenation deteriorated, and such a correlation is more pronounced with LUS scores.

The CT finding seen earliest in COVID-19 pneumonia is ground-glass opacity (GGO). Although located unilaterally in the early stage, GGO is detected in multifocal, bilateral, and dependent regions in the later stages of COVID-19 pneumonia [10]. In advanced stages, the consolidation pattern becomes prominent. The corresponding sonographic findings have been described as B-lines, subpleural, or parenchymal consolidation patterns [7,11]. All sonographic signs detected in our study are very similar to those reported and detected in COVID-19 pneumonia by other studies [7,12,13]. Considering the superficial involvement of COVID-19 pneumonia, we believe that evaluating the pleura and subpleural consolidations is crucial. Therefore, a detailed method for sonographic examination was applied in our study [4,7]. By conducting examinations with both linear and convex probes for LUS, we analyzed and scored both sets of images together. With the linear probe, we identified subpleural consolidated areas and irregularities in the pleural line. A distinctive feature of COVID-19 is the thickening of the pleural line along with irregularities in the pleural line [13]. We also consider that investigating B-line configurations and large consolidated areas with a linear probe is insufficient and necessitates the use of a convex probe.

In many studies, sonography and CT findings for COVID-19 pneumonia have been compared through scoring methods [7,14]. It was found that there were varying degrees of correlation in several studies, and the mean LUS score of the patients varied between 15 and 23 [6,7,12]. In our study, the total LUS score is close to these values. Additionally, in this study, the CO-RADS score shows that most patients had typical CT findings consistent with COVID-19 pneumonia. A weak correlation was found between total LUS scores and total CT scores in total lung areas. In this study, we included patients who underwent CT in the emergency room and had an RT-PCR COVID-19 diagnosis. We then conducted sonographic examinations in the ICU. While the time between these two exams varied from patient to patient, we capped it at 12 hours, mainly due to how our ICU operates. We think that the less-than-ideal correlation between the two types of examinations might be due to this time gap. Ideally, a shorter gap could lead to a stronger correlation, especially given how quickly COVID-19 pneumonia can progress. Furthermore, patients admitted to the ICU were promptly started on respiratory therapy. Some of these patients received noninvasive mechanical ventilation, while others were intubated. It is possible that during this time, positive developments, such as the opening of previously collapsed lung areas in some patients, might have influenced the outcomes.

The LUS score has also been used to classify the severity of the disease. It was found to be greater than the score of 19 in those undergoing mechanical ventilation, and if the score exceeds 23, the disease is classified as severe [6]. The LUS score was found to be 20 in 11 patients undergoing mechanical ventilation. On the other hand, when compared with those receiving noninvasive mechanical ventilation and high-flow oxygen therapy, no difference was detected. Although it seems controversial that there is no difference between the LUS scores of those receiving invasive and noninvasive mechanical ventilation support, respiratory support should not be used as the sole criterion in the evaluation of patients. The reason for such an entity can be considered in a pandemic setting affecting the availability of resources in the ICU [15,16]. At the same time, as the LUS score increased, the oxygenation of our patients also deteriorated. This result was found to be consistent with many other studies [7,12,14]. We also determined that the correlation between CT score and PaO_2_/FiO_2_ ratio was lower. The minor discrepancy in the timing of CT and LUS examinations may have contributed to the observed weaker correlation. Thromboembolic clinical pictures are commonly observed among cases of COVID-19 pneumonia [17]. For this reason, venous structures and cardiac examination have also been recommended to be included in several studies [6,18,19]. As a limitation of our study, additional cardiac and venous structure examinations were not performed. Additional limitations of this study include the non-simultaneous nature of the CT and LUS examinations and the relatively small sample size of our study population.

## Conclusions

This study compared CT findings with LUS results in patients with a confirmed diagnosis of COVID-19 pneumonia. Although the sonographic findings were consistent with COVID-19 pneumonia, only a weak correlation was identified between CT and LUS. It is posited that the limited number of patients and the interval between the sonographic and CT examinations may have influenced the results. Consequently, studies involving a larger patient cohort and synchronous examinations will be instrumental in clarifying this relationship. The correlation of sonographic findings with the level of oxygenation is moderate. These findings suggest that ultrasound may be a useful tool for assessing respiratory failure in critically ill patients.
